# Predicting the Prognosis of Esophageal Adenocarcinoma by a Pyroptosis-Related Gene Signature

**DOI:** 10.3389/fphar.2021.767187

**Published:** 2021-11-18

**Authors:** Ruijie Zeng, Shujie Huang, Xinqi Qiu, Zewei Zhuo, Huihuan Wu, Lei Jiang, Weihong Sha, Hao Chen

**Affiliations:** ^1^ Department of Gastroenterology, Guangdong Provincial People’s Hospital, Guangdong Academy of Medical Sciences, Guangzhou, China; ^2^ Shantou University Medical College, Shantou, China; ^3^ Department of Thoracic Surgery, Guangdong Provincial People’s Hospital, Guangdong Academy of Medical Sciences, Guangzhou, China; ^4^ Zhuguang Community Healthcare Center, Guangzhou, China; ^5^ Guangdong Provincial Geriatrics Institute, Guangdong Provincial People’s Hospital, Guangdong Academy of Medical Sciences, Guangzhou, China

**Keywords:** pyroptosis, esophageal adenocarcinoma, prognosis, methylation, tumor microenvironment

## Abstract

Esophageal adenocarcinoma (EAC) is a highly malignant type of digestive tract cancers with a poor prognosis despite therapeutic advances. Pyroptosis is an inflammatory form of programmed cell death, whereas the role of pyroptosis in EAC remains largely unknown. Herein, we identified a pyroptosis-related five-gene signature that was significantly correlated with the survival of EAC patients in The Cancer Genome Atlas (TCGA) cohort and an independent validation dataset. In addition, a nomogram based on the signature was constructed with novel prognostic values. Moreover, the downregulation of *GSDMB* within the signature is notably correlated with enhanced DNA methylation. The pyroptosis-related signature might be related to the immune response and regulation of the tumor microenvironment. Several inhibitors including GDC-0879 and PD-0325901 are promising in reversing the altered differentially expressed genes in high-risk patients. Our findings provide insights into the involvement of pyroptosis in EAC progression and are promising in the risk assessment as well as the prognosis for EAC patients in clinical practice.

## Introduction

Esophageal cancer is one of the most common malignancies worldwide, accounting for approximately 604,100 new cases and 544,076 deaths per year over the world (Sung et al., 2021). Esophageal adenocarcinoma (EAC) and esophageal squamous cell carcinoma (ESCC) composite the principle histologic subtypes of esophageal cancer, in which the incidence of EAC in western countries has increased dramatically in the last decades (Klingelhöfer et al., 2019). Despite therapeutic advances in surgery, radiotherapy, chemotherapy, and targeted drugs, the 5-year survival of esophageal cancer remains less than 20% (Alsop and Sharma, 2016). In consequence, biomarkers and effective models are urgently needed to predict the prognosis of EAC and provide insights into targeted therapy.

Pyroptosis is a proinflammatory form of regulated cell death, relying on the enzymatic activity of inflammatory proteases that belong to the caspase family (Vande Walle and Lamkanfi, 2016). Pyroptosis is featured with swift plasma-membrane rupture and subsequent release of proinflammatory intracellular contents, which is distinct from apoptosis (Bergsbaken et al., 2009). Studies evaluating the role of pyroptosis in neurological, infectious, autoimmune, cardiovascular, and oncologic disorders have been emerging in recent years (Yu et al., 2021). Activation of the canonical inflammasome pathway is the basis of pyroptosis, in which pattern-recognition receptors (PRRs), for example, Toll-like receptors (TLRs), nucleotide-binding oligomerization domain-like receptors (NLRs), and absent in melanoma 2 like-receptors (ALRs) recognize pathogen-associated molecular patterns (PAMPs) or nonpathogen-related damage-associated molecular patterns (DAMPs) to activate inflammasomes and facilitate caspase-1 activation (Xia et al., 2019). Direct activation of caspase-4/5/11 under lipopolysaccharide (LPS) is involved in the noncanonical pyroptosis pathway, which is independent of the inflammasome complex (Shi et al., 2014). The gasdermin (GSDM) family proteins serve as the main mediators of pyroptosis, which are proteolytically activated by proteases and induce the formation of plasma membrane pores, leading to cell swelling and lysis (Van Opdenbosch and Lamkanfi, 2019; Tsuchiya, 2020). Due to the pivotal role of GSDM family proteins, pyroptosis is defined by some researchers as gasdermin-mediated programmed cell death (Shi et al., 2017).

However, despite the fact that research is emerging in ESCC, the role of pyroptosis in esophageal cancer remains largely unknown, and none of the previous publications have comprehensively evaluated the pyroptosis-related genes in EAC. Therefore, we performed a comprehensive evaluation of pyroptosis-related genes in EAC, in order to develop a pyroptosis-gene-based modality to predict the prognosis of the patients, and provide insights into the correlations between pyroptosis and tumor immune microenvironment.

## Materials and Methods

### Datasets

The RNA-sequencing (RNA-seq) data of 87 patients (78 with EAC; 9 normal samples) and the corresponding clinical information from The Cancer Genome Atlas (TCGA) database were retrieved on May 20, 2021 (https://portal.gdc.cancer.gov/repository). The DNA microarray and clinical features of the validation cohort were downloaded from the Gene Expression Omnibus (GEO) database (https://www.ncbi.nlm.nih.gov/geo/, ID: GSE13898). The initial inclusion criteria were as follows: 1) patients with EAC; 2) patients with clear data for overall survival and survival status; and 3) patients with available gene expression data. The exclusion criteria were as follows: 1) patients with ESCC; 2) patients with incomplete data for overall survival, survival status; and 3) patients without gene expression data. As described in the following sections, further analysis based on clinicopathological characteristics was performed in patients with complete clinical data including age, gender, and stage. Patients with survival time of less than 30 days were excluded.

### Identification of Differentially Expressed Genes in Pyroptosis-Related Gene Set

The 58 pyroptosis-related genes were derived from prior literature and the Gene Ontology (GO) term pyroptosis (ID: GO0070269; Supplementary Table S1) (Man and Kanneganti, 2015; Wang and Yin, 2017; Karki and Kanneganti, 2019; Xia et al., 2019). The microarray data from the GSE13898 cohort were normalized using the quantile normalization method, and the expression levels of genes were transformed to a log_2_ base for further analysis (Kim et al., 2010). The package “limma” was used to explore DEGs with the threshold of *p* value <0.05 (Ritchie et al., 2015). Probes with missing information for gene expressions in >20% samples were removed. The correlations of selected genes were evaluated by the “ggcorrplot” package (Kassambara and Kassambara, 2019). Protein–protein interaction (PPI) networks were created by Search Tool for the Retrieval of Interacting Genes (STRING) and the “igraph” package (Csardi and Nepusz, 2006; Szklarczyk et al., 2019).

### Development and Validation of the Pyroptosis-Correlated Gene Prediction Model for Prognosis

Cox regression analysis was employed to evaluate the value of pyroptosis-related genes for prognosis. The DEGs were identified for further analysis. The LASSO Cox regression analysis was employed to construct a refined model for prognosis using the R package “glmnet” (Friedman et al., 2010). The calculation of the risk score was performed using the following formula: risk score = $\overset{i}{\underset{\left(n=1\right)}{\sum }}Coef\;i*Xi$ (Coef i indicates the coefficient, and Xi indicates the gene expression levels after standardization). The EAC patients were classified into low- and high-risk groups based on the median risk score, and Kaplan–Meier analysis was used to compare the overall survival (OS) between the two groups. Principal component analysis (PCA) was used to assess the separability of the two groups by the “prcomp” function. The R packages “survival,” “survminer,” “timeROC,” and “riskRegression” were utilized for receiver operating characteristic (ROC) curve graphing and area under curve (AUC) calculation for 1, 2, and 5 years (Blanche et al., 2013; Therneau and Lumley, 2015; Kassambara et al., 2017; Ozenne et al., 2017). A nomogram model with clinical features including stage and risk score was constructed by the R packages “rms,” “foreign,” and “survival” (Therneau and Lumley, 2015; Harrell et al., 2017; Team et al., 2020). The calibration curve and detrended correspondence analysis (DCA) were performed using the “rms” package (Harrell et al., 2017). An EAC cohort (GSE13898) from the GEO database was used for validation, and the risk score was calculated by the same methods described above to divide the cohort into two subgroups (low risk and high risk).

### Prognostic Analysis of the Variables

Clinical data (age, gender, and stage) were extracted from patients in the TCGA and GSE13898 cohorts. The clinicopathological characteristics of EAC patients with complete data for further analysis were described in Supplementary Table S2. Variables including gender, stage, and risk score were analyzed in the regression model by univariate and multivariate Cox regression analysis.

### Methylation Analysis

For the genes included in the signature, the cBio Cancer Genomics Portal (cBioPortal) database (http://www.cbioportal.org/) was used for exploring the correlation between methylation alterations and gene expressions in the TCGA esophageal adenocarcinoma cohort. The MEXPRESS database (http://mexpress.be/) was utilized for further assessment of the correlation between the precise genomic location of DNA methylation and altered levels of gene expression. *p* < 0.05 and *R* ^2^ > 0.25 were considered as significant correlation.

### Tumor Microenvironment Analysis

The Tumor Immune Estimation Resource (TIMER) database (https://cistrome.shinyapps.io/timer/) was utilized to assess the correlation between tumor-infiltrating immune cells and expressions of selected genes (Li et al., 2017). Estimation Resource (TIMER) was used to compare the immune scores of the four subtypes. The CIBERSORT algorithm was used to further explore the composition and differences in the fraction of 22 immune cell types between two subgroups classified by risk scores (Chen et al., 2018).

### Enrichment Analysis

Patients with EAC in the TCGA cohort were divided into two groups based on the median risk score. The DEGs between the low- and high-risk groups were extracted by |log2FC| ≥ 1 and *p* value <0.05. GO and Kyoto Encyclopedia of Genes and Genomes (KEGG) pathway enrichment was performed by the R package “clusterProfiler,” and the results were visualized using the “GOplot” package (Yu et al., 2012; Walter et al., 2015).

### Connectivity Map Analysis

CMap analysis was performed and visualized in https://clue.io/(Lamb et al., 2006), The top 150 upregulated and downregulated genes were selected according to the |logFC| values of DEGs for CMap analysis to identify a shortlist of drugs. According to the pattern-matching algorithms, positive scores indicate the induction effect of the small molecules on the signature, while negative scores indicate the inhibition effect. The drugs were further selected based on the negative scores.

### Statistical Analysis

Statistical analyses were performed by R (version 4.1.0). Student’s t-test was applied to compare the differences in gene expression between tumor and normal tissues, while categorical variables were compared using Pearson's chi-square test. The OS of patients between low- and high-risk groups were compared by the Kaplan–Meier method with log-rank test. The Cox regression analysis was performed to evaluate the independent prognostic factors for survival. The Wilcoxon test was used to compare the immune cell infiltration between groups.

### Code Availability

The R code used in this study is available from the corresponding author upon reasonable request.

## Results

### Identification of DEGs Between EAC and Normal Tissues

**FIGURE 1:**
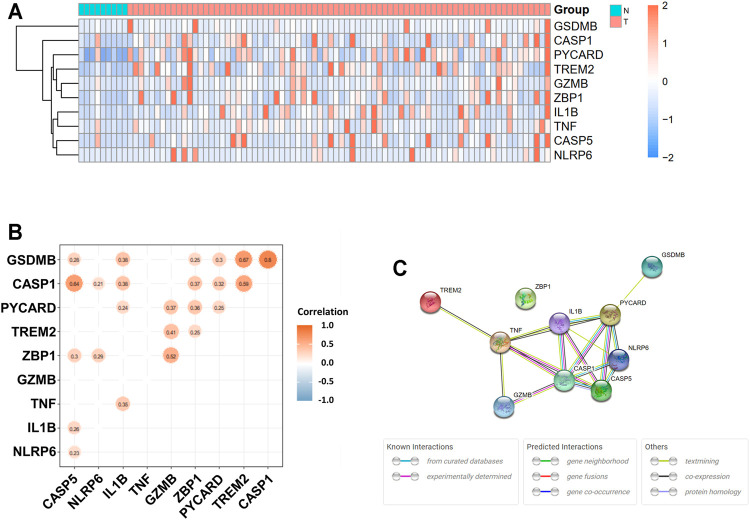
Expressions of 10 differentially expressed pyroptosis-related genes and interaction. **(A)** Heatmap of gene expression between the tumor (T, red color) and normal (N, blue color) groups. Higher expression: red color. Lower expression: blue color. **(B)** Correlation network of 10 differentially expressed pyroptosis-related genes. Nonsignificant correlations (*p* > 0.05) were not shown in the figure. Orange: higher levels of correlation; blue: lower levels of correlation. **(C)** Protein–protein interaction (PPI) network of proteins encoded by selected genes.

The expression levels of 58 pyroptosis-correlated genes were examined in the TCGA data of 78 EAC and 9 normal tissues. Ten DEGs were identified (|log2FC| ≥ 1 and *p* value <0.05), and all of them (*CASP1, CASP5, GSDMB, GZMB, IL1B, NLRP6, PYCARD, TNF, TREM2,* and *ZBP1*) were upregulated in the tumor group. The expression profiles of DEGs were demonstrated in Figure 1A (red color represents a higher expression level; blue color represents a lower expression level). Figure 1B showed the correlation network of DEGs in the TCGA data, indicating that GSDMB expressions are strongly correlated with CASP1 (*r* = 0.80, *p* < 0.05) and TREM2 (*r* = 0.67, *p* < 0.05) expressions. In addition, the expression of CASP1 is significantly correlated with that of CASP5 (*r* = 0.64, *p* < 0.05). The PPIs of DEGs were presented in Figure 1C, in which the interaction score was set as 0.4. The correlation between CASP1 and CASP5 was consistent in the protein level.

### Construction of Prognostic Model Based on DEGs

**FIGURE 2:**
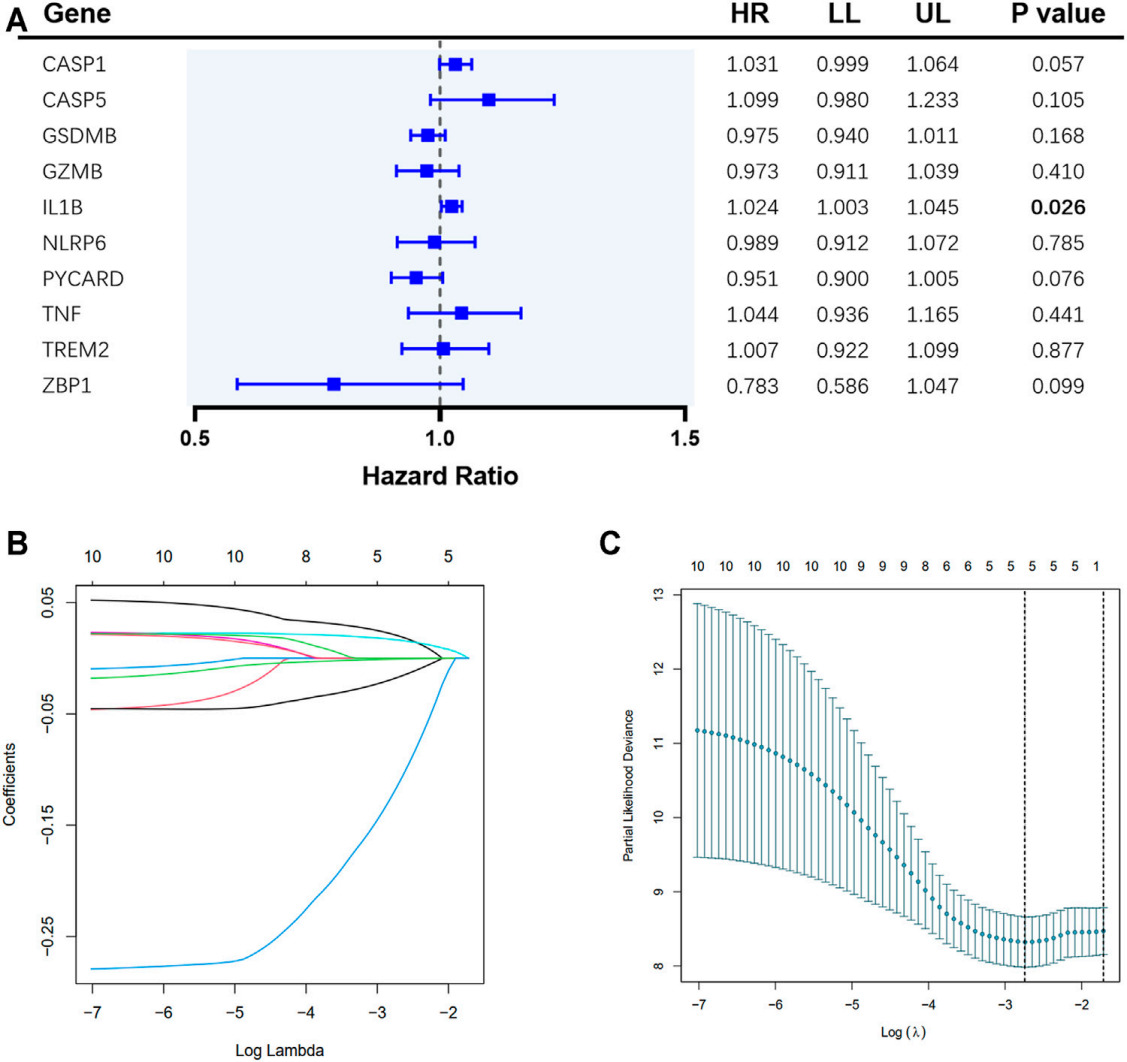
Construction of pyroptosis-related gene risk signature in the TCGA cohort. **(A)** Univariate Cox regression analysis of overall survival (OS) for the selected genes. **(B)** LASSO regression of the 10 selected genes. **(C)** Cross-validation for tuning the parameter selection in LASSO regression.

**FIGURE 3:**
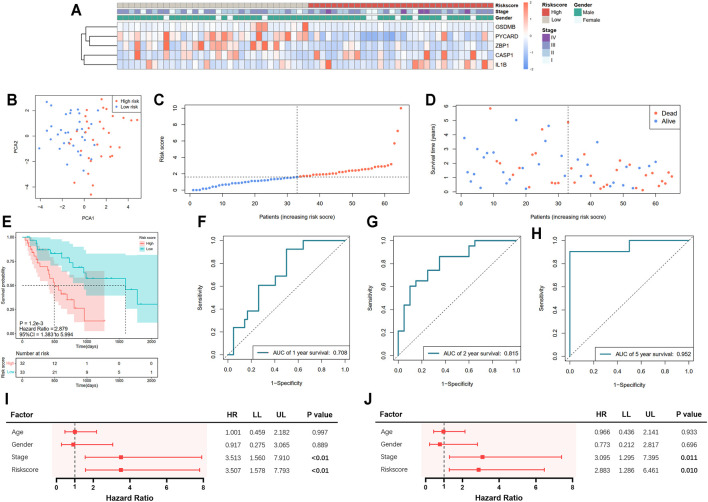
Prediction of prognosis using the pyroptosis-related five-gene signature in the TCGA cohort. **(A)** Heatmap of five selected gene expressions with clinical features ordered by risk score (red: higher expression; blue: lower expression). **(B)** Principal component analysis (PCA) of the risk groups. **(C)** Distribution of patients according to risk scores. **(D)** Survival time and status of patients. **(E)** Kaplan–Meier curves for the survival of patients in the low- and high-risk groups. **(F–H)** Receiver operating characteristic (ROC) curve for 1-, 2-, and 5-year survival of patients. **(I)** Univariate Cox analysis. **(J)** Multivariate Cox analysis.

A total of 65 EAC patients with available survival data were included in our study. Univariate Cox regression analysis was initially performed to assess the prognostic value of DEGs (Figure 2A). Among them, six genes (*CASP1, CASP5, GSDMB, IL1B, PYCARD*, and *ZBP1*) were with *p* value <0.2, and higher expressions of *CASP1, CASP5,* and *IL1B* were associated with increased risk (HR > 1), while upregulated expressions of *GSDMB, PYCARD,* and *ZBP1* were correlated with lower risk (HR < 1). Subsequently, LASSO Cox regression analysis retrieved five genes for prognostic model construction based on the optimum *λ* value (Figures 2B,C). The calculation of the risk score was as follows: Risk score = (0.042 × exp*CASP1*) + (−0.025 × exp*GSDMB*) + (0.021 × expIL1B) + (−0.037 × exp*PYCARD*) + (−0.243 × exp*ZBP1*). According to the calculated median risk score, 65 patients were divided into two groups (32 in the high-risk group and 33 in the low-risk group), and the clinical information is shown in Figure 3A. The PCA illustrated that patients were well divided into two clusters (Figure 3B). The distributions of the risk score and survival time are shown in Figures 3C,D. The OS of the high-risk group was significantly worse than that of the low-risk group (*p* = 0.0012, Figure 3E). ROC analysis of the risk model indicated that the AUC for 1, 2, and 5-year survival was 0.708, 0.815, and 0.952, respectively (Figures 3F–H). Both of the univariate and multivariate Cox regression analyses showed that the pyroptosis-related gene signature independently predicted the prognosis of EAC patients (Figures 3I,J).

### Verification of the Gene Signature by the External Dataset

**FIGURE 4:**
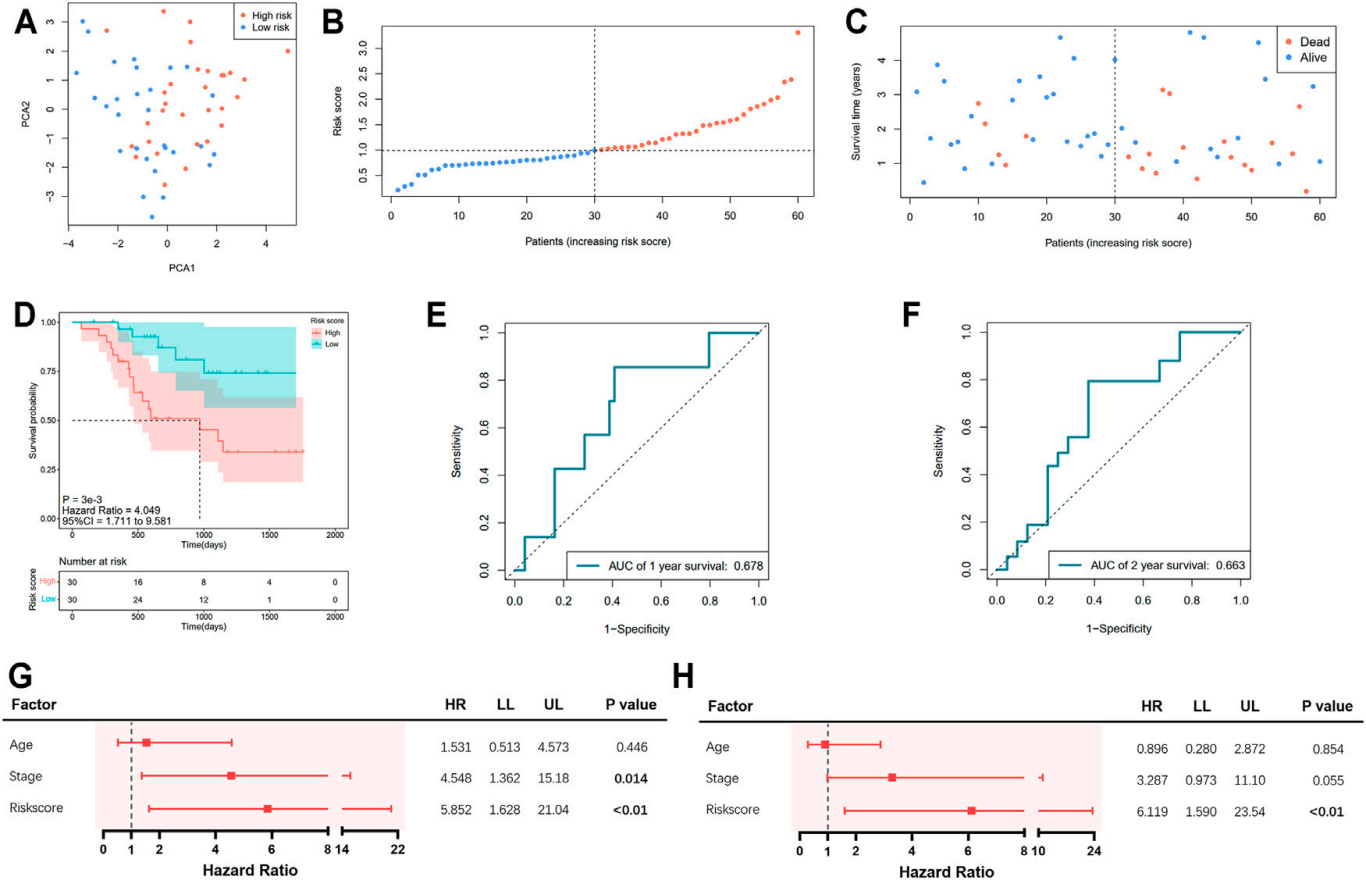
Validation of the pyroptosis-related five-gene signature in the GSE13898 cohort. **(A)** Principal component analysis (PCA) of the risk groups. **(B)** Distribution of patients according to risk scores. **(C)** Survival time and status of patients. **(D)** Kaplan–Meier curves for the survival of patients in the low- and high-risk groups. **(E, F)** Receiver operating characteristic (ROC) curve for 1-, 2-, and 5-year survival of patients. **(G)** Univariate Cox analysis. **(H)** Multivariate Cox analysis.

Information of 60 EAC patients from the GSE13898 dataset of GEO with available survival data was used for the validation of the gene signature. The expressions of the available differentially expressed pyroptosis-related genes are shown in Supplementary Figure S1. The patients were subdivided into the low- and high-risk groups, respectively, as described above. PCA illustrated well the separation of patients between the two groups (Figure 4A). The distribution of the risk score and the survival time is demonstrated in Figures 4B,C. Patients in the low-risk group were with significantly higher survival rates than those in the high-risk group (*p* = 0.003; Figure 4D). According to the ROC curve, the 1- and 2-year survival prediction models were with AUCs of 0.678 and 0.663 (Figures 4E,F), respectively, while the 5-year survival prediction model could not be generated due to insufficient data. The risk score in our model could also serve as an independent prognostic factor in the validation cohort (Figures 4G,H).

### Construction of Nomogram Based on the Gene Signature and Clinical Data

**FIGURE 5:**
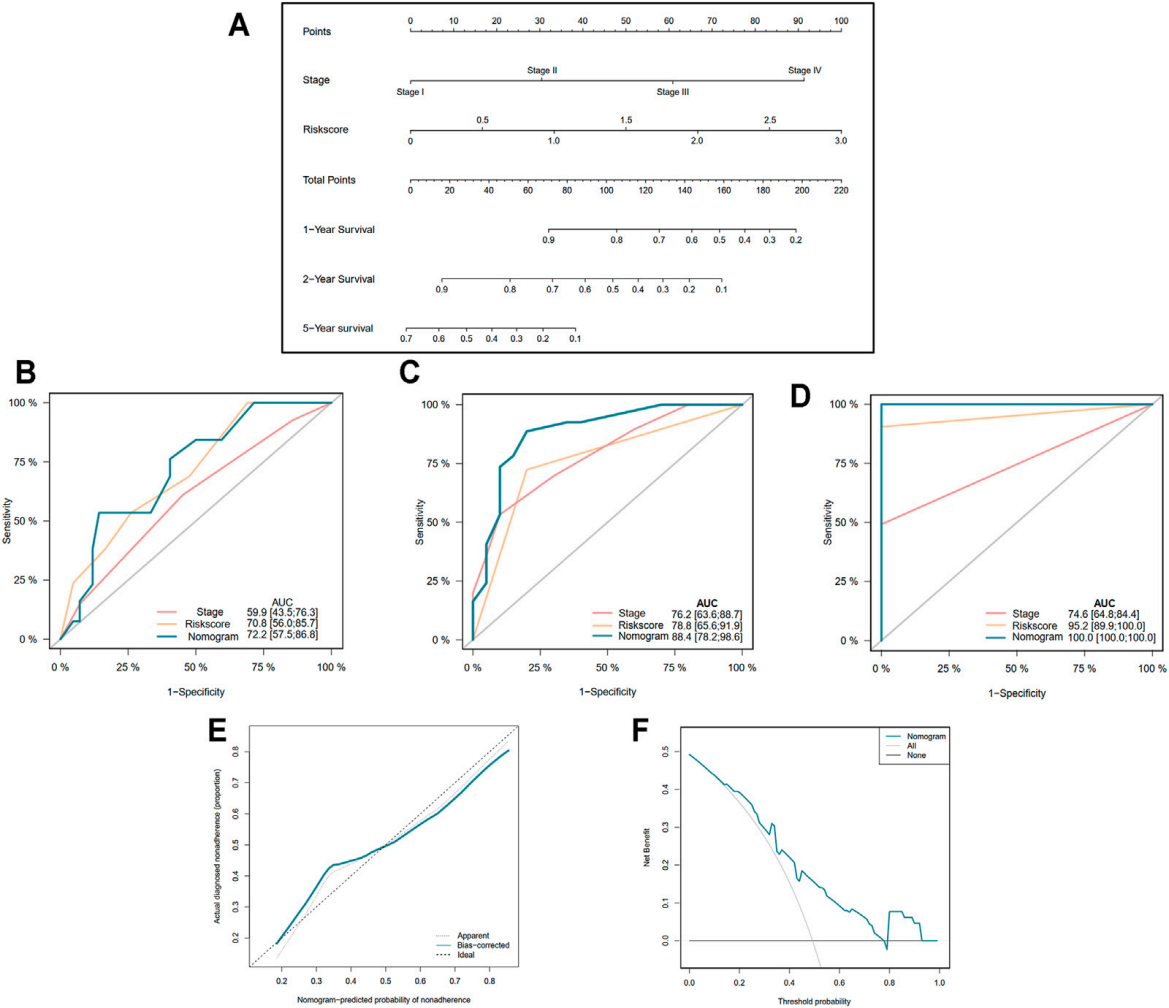
Construction of nomogram based on the pyroptosis-related five-gene signature. **(A)** Nomogram for predicting 1-, 2-, and 5-year survival of EAC patients. **(B–D)** Receiver operating characteristic (ROC) curve evaluating the efficiency of nomogram for 1-, 2-, and 5-year survival of patients. **(E)** Calibration curve of nomogram. **(F)** Decision curve analysis (DCA) curve of the nomogram.

In order to more precisely predict the prognosis of EAC patients, the TNM stage was used to construct a nomogram model as shown in Figure 5A (C-index = 0.764 ± 0.046). The AUCs of the nomogram for predicting 1-, 2-, and 5-year survival were 0.722, 0.884, and 1.000, respectively (Figures 5B–D). The calibration curve indicated an ideal prediction of the nomogram (Figure 5E). Figure 5F shows that when the nomogram-predicted probability ranged from 15% to 80%, the nomogram provided extra value relative to the treat-all-patients scheme or the treat-none scheme.

### The Expressions of GSDMB and ZBP1 Within the Signature Are Downregulated by Hypermethylation

**FIGURE 6:**
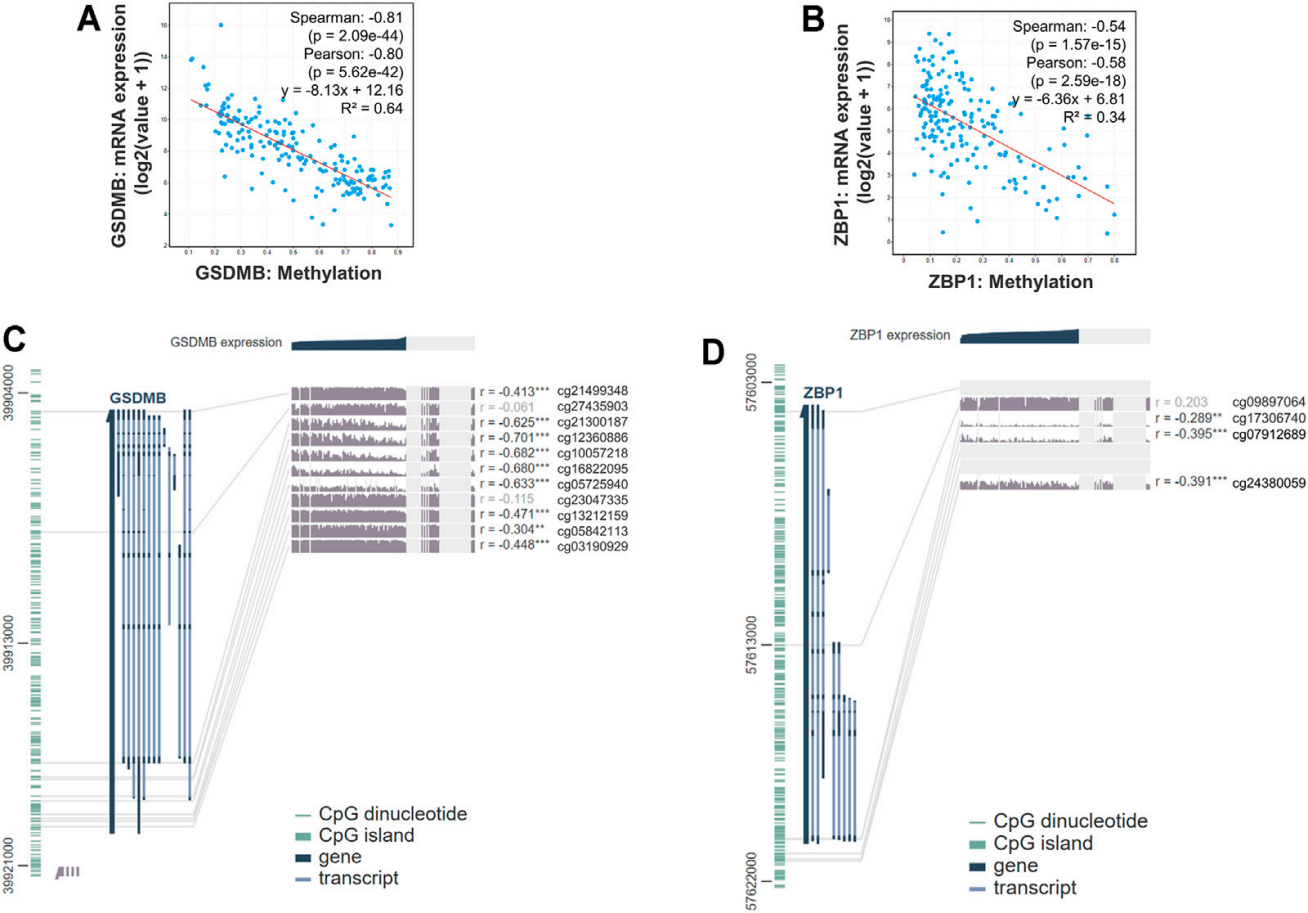
Gene expression and methylation status in EAC. The correlation between *GSDMB* **(A)** and *ZBP1* **(B)** methylation with RNA expressions by analysis using the cBioPortal database. Detailed information on methylated probes of *GSDMB* **(C)** and *ZBP1* **(D)** by analysis using the MEXPRESS database.

Epigenetic regulations including DNA methylation affect gene expression and modulate various cellular responses in tumorigenesis. Therefore, we further explored the mechanisms that might be involved in controlling the expressions of genes involved in the signature. We found that the RNA expressions of *GSDMB* (Spearman: −0.81, *p* = 2.09e-44; Pearson: −0.80, *p* = 5.62e-42, *R* ^2^ = 0.64) and *ZBP1* (Spearman: −0.54, *p* = 1.57e-15; Pearson: −0.58, *p* = 2.59e-18, *R* ^2^ = 0.34) were significantly correlated with the DNA methylation status (Figures 6A,B), whereas the association between RNA expressions of *CASP1, IL1B*, and *PYCARD* and DNA methylation was nonsignificant (Supplementary Figure S2). Analysis by the MEXPRESS database further identified the detailed information of the methylated probes and their correlation with *GSDMB* (Figure 6C) and *ZBP1* (Figure 6D) RNA expressions, suggesting that the expressions of *GSDMB* and *ZBP1* could be regulated by epigenetic mechanisms.

### Differential Expression Analysis Reveals Immune-Correlated Pathways

**FIGURE 7:**
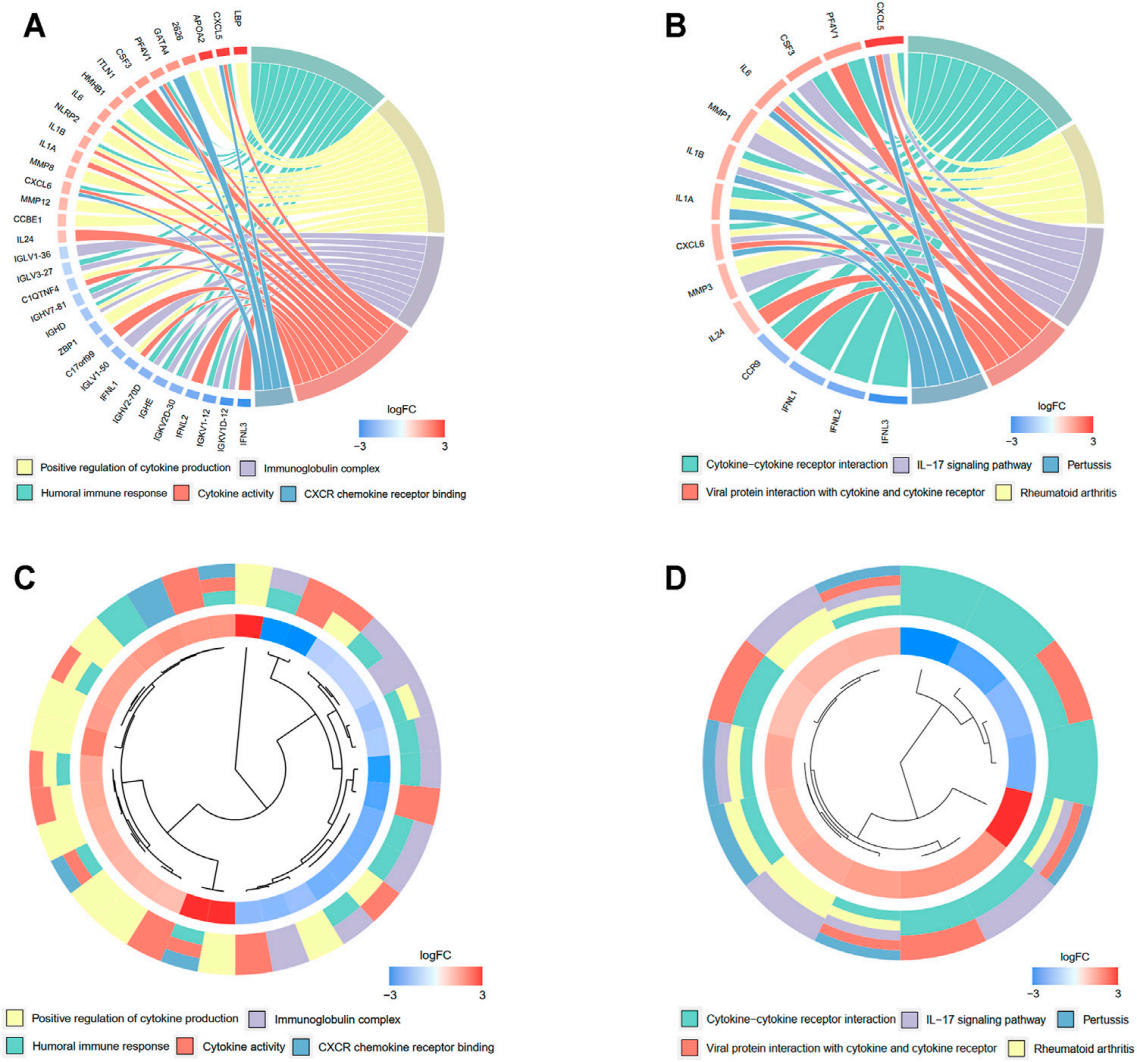
Functional enrichment analysis of differentially regulated genes (DEGs) between low- and high-risk groups. **(A, B)** Chord plot demonstrating the linkage of genes and assigned term by GO **(A)** and KEGG **(B)** pathway enrichment analysis. **(C, D)** Cluster plot of the expression profiles. The inner ring shows the color-coded logFC, while the outer ring illustrates the assigned functional terms by GO **(C)** and KEGG **(D)** pathway enrichment analysis.

A total of 527 DEGs between the low- and high-risk groups were extracted according to the threshold described above. A total of 310 genes were downregulated in the high-risk group, while 217 genes were upregulated in the low-risk group. On the basis of the DEGs, GO enrichment and KEGG pathway analyses were performed. The results from GO enrichment analysis demonstrated that the DEGs were mainly associated with the regulation of cytokine production, cytokine activity, and humoral immune response pathways (Figures 7A,B) in the TCGA cohort. KEGG pathway analysis showed that the DEGs were principally associated with the cytokine–cytokine receptor interaction and IL-17 signaling pathways (Figures 7C,D) in the TCGA cohort. Detailed information for the deregulated pathways is shown in Supplementary Figures S3A–D. Similarly, the GO analysis in the GSE13898 cohort demonstrated that immune-related pathways, including neutrophil activation for immune response, neutrophil-mediated immunity, and cytokine and chemokine receptor binding pathways, were deregulated between the two groups divided by the risk score (Supplementary Figures S4A, 3B). The DEGs in the GSE13898 cohort were also associated with the IL-17 signaling pathway (Supplementary Fig. S4C, 3D).

### Pyroptosis-Related Gene Signature Is Related to the Immune Status of EAC

The CMap analysis was performed to screen for small-molecular drugs that are able to revert the pyroptosis signature-related pathways, which contribute to a high-risk state. A total of 730 drugs with negative scores were identified (Supplementary Table S3). The RAF inhibitor GDC-0879 (score: −93.76), mitogen-activated protein kinase kinase (MEK) inhibitor PD-0325901 (score: −91.95), heat shock protein (HSP) inhibitor VER-155008 (score: −89.75), mitogen-activated protein (MTOR) inhibitor torin-2 (score: −87.13), and aryl hydrocarbon receptor ligand GR-206 (score: −86.62) were the top five small-molecular drugs based on inhibition scores (Table 1).

**TABLE 1:** List of the five most significant small molecular compounds to potentially reverse altered expression of differentially expressed genes (DEGs) in the high-risk group.

Name	Score	Description	Target	MOA
GDC-0879	−93.76	RAF inhibitor	BRAF	RAF inhibitor
PD-0325901	−91.95	MEK inhibitor	MAP2K1, MAP2K2	MEK inhibitor, MAP kinase inhibitor, Protein kinase inhibitor
VER-155008	−89.75	HSP inhibitor	HSPA1A	HSP inhibitor
torin-2	−87.13	MTOR inhibitor	MTOR	MTOR inhibitor
GR-206	−86.62	Aryl hydrocarbon receptor ligand	—	Aryl hydrocarbon receptor ligand

MOA, mechanisms of action; MEK, mitogen-activated protein kinase kinase; MAP, mitogen-activated protein; HSP, heat shock protein; MTOR, mammalian target of rapamycin.

### Pyroptosis-Related Gene Signature Is Related to the Immune Status of EAC

**FIGURE 8:**
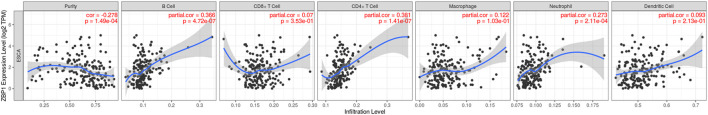
Correlations between immune cells and selected genes. ZBP1 and immune cells in esophageal cancer.

To explore the correlation between the selected pyroptosis-related genes and gene-based signature with the immune microenvironment of EAC, analysis by the TIMER database for each gene was initially performed. The results indicated that *ZBP1* expression was most significantly correlated with the infiltration signature of esophageal cancer, in which infiltrations of B cells (correlation coefficient = 0.366, *p* = 4.72e-07) and CD4^+^ T cells (correlation coefficient = 0.381, *p* = 1.41e-07) were with the most remarkable correlations (Figure 8A, Supplementary Figure S5). In addition, somatic copy number alterations of *ZBP1* were correlated with the infiltration levels of B cells, CD8^+^ T cells, macrophages, and dendritic cells (Figure 8B).

**FIGURE 9:**
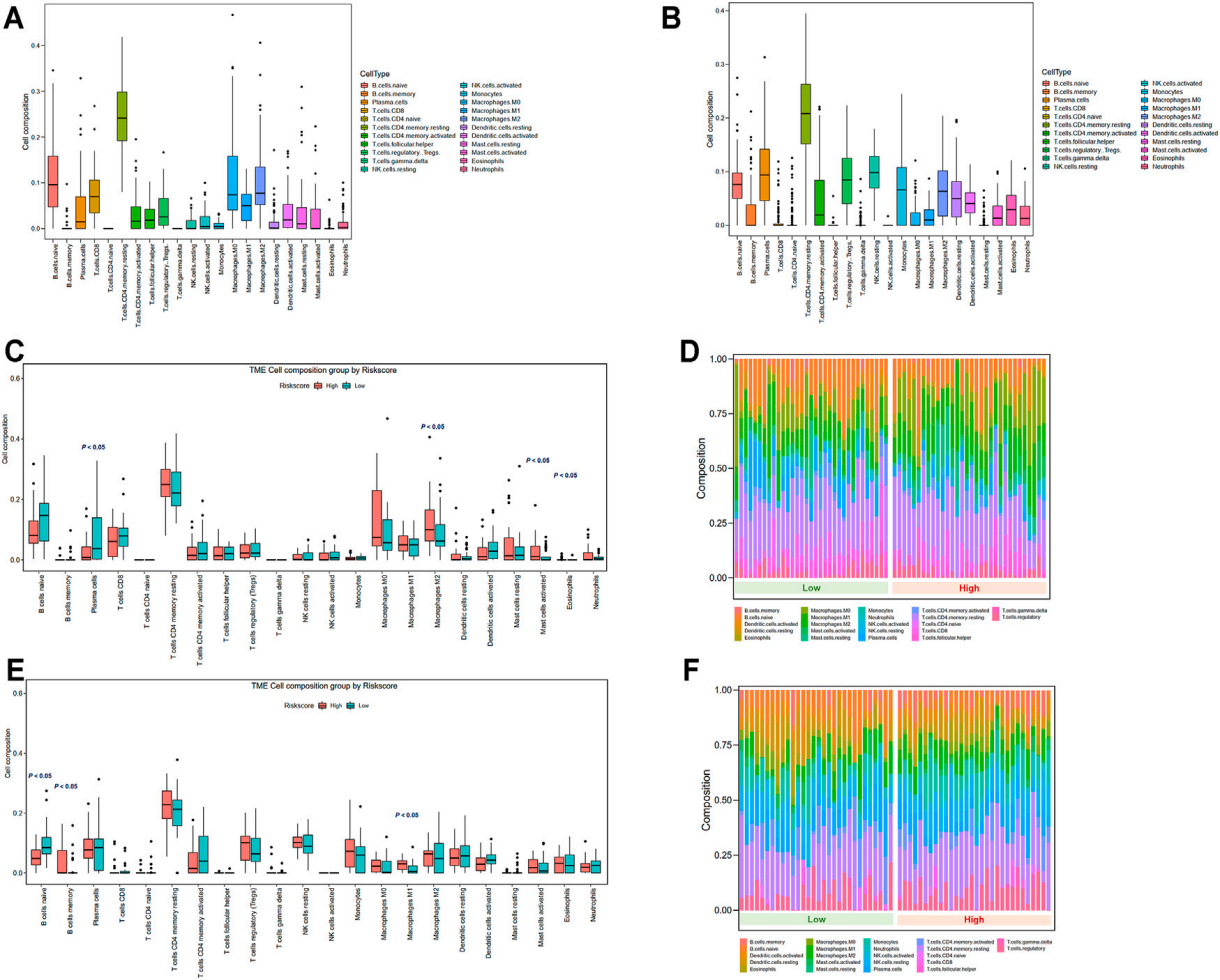
Tumor microenvironment immune cell composition in low- and high-risk groups. **(A)** Overview of immune cell composition in the TCGA cohort. **(B)** Overview of immune cell composition in the GSE13898 cohort. **(C, D)** Differences in immune cell composition in the TCGA cohort classified by risk groups by Wilcoxon test. **(E, F)** Differences in immune cell composition in the GSE13898 cohort classified by risk groups by Wilcoxon test.

The variations in the abundance of immune cell infiltration between low- and high-risk groups were further explored. The immune cells were analyzed in the TCGA (Supplementary Table S4) and GSE13898 cohorts (Supplementary Table S5). The overview of immune cell compositions is illustrated in Figure 9A for the TCGA cohort and in Figure 9B for the GSE13898 cohort. The high-risk group in the TCGA cohort possessed significantly higher infiltration levels of M2 macrophages, activated mast cells, and eosinophils, whereas the infiltration levels of plasma cells were significantly lower (Figures 9C,D). By contrast, the infiltration levels of memory B cells and M1 macrophages were upregulated in the high-risk group of the GSE13898 cohort, while those of naïve B cells were significantly downregulated (Figures 9E,F).

## Discussion

Cell death serves as an essential barrier against the development of cancer, and pyroptosis is one of the major forms of programmed cell death (Bertheloot et al., 2021). However, the role of pyroptosis in EAC remains largely unclear. In the present study, we comprehensively evaluated the pyroptosis-related gene profiles in EAC and constructed a novel five-gene risk signature (*CASP1, GSDMB, IL1B, PYCARD,* and *ZBP1*) by LASSO Cox regression analysis. The five-gene signature showed good performance for predicting EAC prognosis in both the internal and external validation cohorts. Within the signature, *GSDMB* expression is distinctly correlated with the methylation status. Further enrichment analyses revealed that the DEGs between the low- and high-risk groups were correlated with immune-related pathways. The RAF inhibitor GDC-0879 and the MEK inhibitor PD-0325901 might be promising in reverting the pyroptosis-related pathways in the high-risk EAC patients. Tumor immune microenvironment analyses indicated that high-risk patients had decreased levels of infiltrating active immune cells and higher proportions of quiescent immune-cell infiltration.

For the components within the signature, Gasdermin B (GSDMB) belongs to the GSDM family and is more broadly expressed compared to other GSDM family members (Saeki et al., 2009). The cleavage of GSDMB induced by lymphocyte-derived granzyme A triggers pyroptosis (Zhou et al., 2020). Therefore, the downregulation of *GSDMB* is associated with poorer prognosis of the patients. Caspase 1 encoded by *CASP1* is a member of the caspase family, which is activated by inflammasomes and induces pyroptosis (Miao et al., 2011). By contrast, caspase 1 can direct T cell-independent tumor proliferation and correlates with a poorer prognosis (Zeng et al., 2018). Interleukin 1 beta (IL-1β) is a proinflammatory cytokine involved in pyroptosis. CASP-1 directly cleaves GSMD and precursor cytokines into pro-IL-1β, which initiates pyroptosis and maturation of IL-1β, respectively (Man et al., 2017). IL-1β has pro-tumorigenic effects by promoting proliferation, migration, metastasis, and angiogenesis (Gelfo et al., 2020; Rébé and Ghiringhelli, 2020). In the present study, *CASP1* and *IL1B* upregulation is associated with a worse prognosis of EAC patients. Apoptosis-associated speck-like protein containing a CARD (ASC/PYCARD) is encoded by the *PYCARD* gene and contains a caspase activation and recruitment domain (CARD) for binding and facilitating the activation of caspase 1 (Bergsbaken et al., 2009). The dual role of the inflammasome adaptor PYCARD is identified in cancer cells, and therefore, PYCARD can be associated with lower cancer risks (Protti and De Monte, 2020). Z-DNA-binding protein 1 (ZBP1)-NLR Family Pyrin Domain Containing 3 (NLRP3) is critical in inducing pyroptosis by leading to cytokine maturation and GSDMD cleavage (Zheng and Kanneganti, 2020). *ZBP1* expression was found to reduce tumor cell motility and chemotaxis, which decreased the potential of metastasis of tumor cells (Lapidus et al., 2007). ZBP1 stabilizes intercellular connections and focal adhesions, which suppresses breast cancer cell invasion (Gu et al., 2012). *PYCARD* and *ZBP1* were identified as downregulated in the high-risk EAC populations. Therefore, inflammasome components might exert different effects in tumor development and progression depending on the biological context, and further investigations are needed.

Epigenetic regulation mechanisms, particularly DNA methylation, modify gene expression and regulate various cellular responses in cancer including proliferation, invasion, apoptosis, and senescence (Cheng et al., 2019). Our study reveals that *GSDMB* promoter hypermethylation most notably induces decreased expression levels, indicating that methylation is essential for the regulation of pyroptosis in EAC. In recent years, epigenetic drugs are emerging, and hundreds of clinical trials are ongoing for investigating the effects of anti-DNA methylation therapies (Cheng et al., 2019). Therefore, our results suggest that epigenetics-targeted therapy is a promising strategy for future anticancer therapeutics in part by modulating the pyroptosis-related genes. Nomograms are promising for use in clinical practice for evaluating the prognosis of EAC patients, in which the survival can be predicted using specific parameters. As indicated by the ROC curves, the nomogram demonstrates high predictive accuracy and sensitivity. Compared to the conventional TNM staging and a previously developed ferroptosis-related gene signature (AUC = 0.744) in EAC (Zhu et al., 2021), the pyroptosis-related gene signature-based nomogram, which integrates gene expression profiles and clinical parameters, more effectively predicts the prognosis of EAC patients. In addition, the prognostic value of our signature is better than the DNA repair-based gene signature (AUC = 0.759) in esophageal cancer (Wang et al., 2021). The prognostic value for 3- and 5-year survival is also higher than a recently developed signature based on nine immune-related genes for esophageal cancer (AUC = 0.826) (Zhang et al., 2021). The use of nomogram based on integrated information can facilitate the prediction of prognosis, clinical decision-making, and patient counseling (Bobdey et al., 2018).

The tumor immune microenvironment is diverge and complex, which contributes to tumorigenesis and modulates the effects of immunotherapy to a large extent. Current studies on lymphocytes in tumor immunity predominantly focus on T cells, while the protective effect of B cells has also been revealed (Wang et al., 2019). By contrast, mast cells have been reported to induce cancer growth (Maciel et al., 2015). Activated T cells, natural killer cells, and macrophages are potent suppressors that mediate the tumor microenvironment and exert antitumor functions (Lin et al., 2013; Nurieva et al., 2019; Cózar et al., 2021). Although some of the comparisons were not statistically different and might be contributed by the limited number of samples in both cohorts, accumulation of immune cells that promote cancer in the tumor microenvironment was generally observed in the high-risk group in both the TCGA and GEO cohorts, while the compositions of tumor-protective immune cells were reduced compared to the low-risk group.

The strength of our study is that a systemic analysis was performed based on the TCGA and GEO cohorts, and the pyroptosis-related genes were assessed for the first time. Limitations also exist in our study. Current publicly available datasets are limited in both number and size, and therefore, validation of our prediction model in large-scale EAC cohorts could be performed in future studies. In addition, based on the information of our study, further *in vitro* and *in vivo* studies could be conducted to evaluate the function and mechanisms of pyroptotic regulation in EAC. Despite the limitations, our study has provided a comprehensive overview of pyroptosis-related gene profiles in EAC.

In summary, we identified differentially expressed pyroptosis-related genes and developed a novel five-gene pyroptosis signature that significantly correlates with the survival of EAC patients. The pyroptosis-based signature is an independent prognostic factor and performs better than the TNM stage, which is promising for clinical application. Moreover, GSDMB expression is notably correlated with methylation status, and the signature is related to antitumor immunity in the tumor microenvironment. Modulating pyroptosis, epigenetic mechanisms, and immune microenvironment by drug discovery might be promising for improving the prognosis of patients. Further studies exploring the regulating patterns are warranted.

## Data Availability

The datasets presented in this study can be found in online repositories. The names of the repository/repositories and accession number(s) can be found in the article/Supplementary Material.

## References

[B1] Alsop B. R. Sharma P. (2016). Esophageal Cancer. Gastroenterol. Clin. North. Am. 45, 399–412. 10.1016/j.gtc.2016.04.001 27546839

[B2] Bergsbaken T. Fink S. L. Cookson B. T. (2009). Pyroptosis: Host Cell Death and Inflammation. Nat. Rev. Microbiol. 7, 99–109. 10.1038/nrmicro2070 19148178 PMC2910423

[B3] Bertheloot D. Latz E. Franklin B. S. (2021). Necroptosis, Pyroptosis and Apoptosis: an Intricate Game of Cell Death. Cell Mol Immunol 18, 1106–1121. 10.1038/s41423-020-00630-3 33785842 PMC8008022

[B4] Blanche P. Dartigues J. F. Jacqmin-Gadda H. (2013). Estimating and Comparing Time-dependent Areas under Receiver Operating Characteristic Curves for Censored Event Times with Competing Risks. Stat. Med. 32, 5381–5397. 10.1002/sim.5958 24027076

[B5] Bobdey S. Mair M. Nair S. Nair D. Balasubramaniam G. Chaturvedi P. (2018). A Nomogram Based Prognostic Score that Is superior to Conventional TNM Staging in Predicting Outcome of Surgically Treated T4 Buccal Mucosa Cancer: Time to Think beyond TNM. Oral Oncol. 81, 10–15. 10.1016/j.oraloncology.2018.04.002 29884407

[B6] Chen B. Khodadoust M. S. Liu C. L. Newman A. M. Alizadeh A. A. (2018). “Profiling Tumor Infiltrating Immune Cells with CIBERSORT,” in Cancer Systems Biology (Clifton, NJ: Springer), 243–259. 10.1007/978-1-4939-7493-1_12 PMC5895181 29344893

[B7] Cheng Y. He C. Wang M. Ma X. Mo F. Yang S. (2019). Targeting Epigenetic Regulators for Cancer Therapy: Mechanisms and Advances in Clinical Trials. Signal. Transduct Target. Ther. 4, 62–39. 10.1038/s41392-019-0095-0 31871779 PMC6915746

[B8] Cózar B. Greppi M. Carpentier S. Narni-Mancinelli E. Chiossone L. Vivier E. (2021). Tumor-infiltrating Natural Killer Cells. Cancer Discov. 11, 34–44. 10.1158/2159-8290.CD-20-0655 33277307 PMC7611243

[B9] Csardi G. Nepusz T. (2006). The Igraph Software Package for Complex Network Research. InterJournal, complex Syst. 1695, 1–9.

[B10] Friedman J. Hastie T. Tibshirani R. (2010). Regularization Paths for Generalized Linear Models *via* Coordinate Descent. J. Stat. Softw. 33, 1–22. 10.18637/jss.v033.i01 20808728 PMC2929880

[B11] Gelfo V. Romaniello D. Mazzeschi M. Sgarzi M. Grilli G. Morselli A. (2020). Roles of IL-1 in Cancer: From Tumor Progression to Resistance to Targeted Therapies. Int. J. Mol. Sci. 21, 6009. 10.3390/ijms21176009 PMC7503335 32825489

[B12] Gu W. Katz Z. Wu B. Park H. Y. Li D. Lin S. (2012). Regulation of Local Expression of Cell Adhesion and Motility-Related mRNAs in Breast Cancer Cells by IMP1/ZBP1. J. Cel Sci 125, 81–91. 10.1242/jcs.086132 PMC3269024 22266909

[B13] Harrell F. E. Jr Harrell M. F. E. Jr Hmisc D. (2017). Package ‘rms’. Vanderbilt University 229. Available at: http://CRAN.R-project.org/package = rms 2017.

[B14] Karki R. Kanneganti T. D. (2019). Diverging Inflammasome Signals in Tumorigenesis and Potential Targeting. Nat. Rev. Cancer 19, 197–214. 10.1038/s41568-019-0123-y 30842595 PMC6953422

[B15] Kassambara A. Kassambara M. A. (2019). Package ‘ggcorrplot’. *R Package.* version 0.1 3. Available at: https://cran.r-project.org/package=ggcorrplot.

[B16] Kassambara A. Kosinski M. Biecek P. Fabian S. (2017). Survminer: Drawing Survival Curves Using'ggplot2. R. Package Version 0.3 1. Available at: https://cran.r-project.org/web/packages/ggplot2.

[B17] Kim S. M. Park Y. Y. Park E. S. Cho J. Y. Izzo J. G. Zhang D. (2010). Prognostic Biomarkers for Esophageal Adenocarcinoma Identified by Analysis of Tumor Transcriptome. PloS one 5, e15074. 10.1371/journal.pone.0015074 21152079 PMC2994829

[B18] Klingelhöfer D. Zhu Y. Braun M. Brüggmann D. Schöffel N. Groneberg D. A. (2019). A World Map of Esophagus Cancer Research: a Critical Accounting. J. Transl Med. 17, 150. 10.1186/s12967-019-1902-7 31077194 PMC6511204

[B19] Lamb J. Crawford E. D. Peck D. Modell J. W. Blat I. C. Wrobel M. J. (2006). The Connectivity Map: Using Gene-Expression Signatures to Connect Small Molecules, Genes, and Disease. science 313, 1929–1935. 10.1126/science.1132939 17008526

[B20] Lapidus K. Wyckoff J. Mouneimne G. Lorenz M. Soon L. Condeelis J. S. (2007). ZBP1 Enhances Cell Polarity and Reduces Chemotaxis. J. Cel Sci. 120, 3173–3178. 10.1242/jcs.000638 PMC4956933 17878234

[B21] Li T. Fan J. Wang B. Traugh N. Chen Q. Liu J. S. (2017). TIMER: A Web Server for Comprehensive Analysis of Tumor-Infiltrating Immune Cells. Cancer Res. 77, e108–e110. 10.1158/0008-5472.CAN-17-0307 29092952 PMC6042652

[B22] Lin Y. C. Mahalingam J. Chiang J. M. Su P. J. Chu Y. Y. Lai H. Y. (2013). Activated but Not Resting Regulatory T Cells Accumulated in Tumor Microenvironment and Correlated with Tumor Progression in Patients with Colorectal Cancer. Int. J. Cancer 132, 1341–1350. 10.1002/ijc.27784 22907255

[B23] Maciel T. T. Moura I. C. Hermine O. (2015). The Role of Mast Cells in Cancers. F1000prime Rep. 7, 09. 10.12703/P7-09 25705392 PMC4311277

[B24] Man S. M. Kanneganti T. D. (2015). Regulation of Inflammasome Activation. Immunol. Rev. 265, 6–21. 10.1111/imr.12296 25879280 PMC4400844

[B25] Man S. M. Karki R. Kanneganti T. D. (2017). Molecular Mechanisms and Functions of Pyroptosis, Inflammatory Caspases and Inflammasomes in Infectious Diseases. Immunol. Rev. 277, 61–75. 10.1111/imr.12534 28462526 PMC5416822

[B26] Miao E. A. Rajan J. V. Aderem A. (2011). Caspase-1-induced Pyroptotic Cell Death. Immunol. Rev. 243, 206–214. 10.1111/j.1600-065X.2011.01044.x 21884178 PMC3609431

[B27] Nurieva R. I. Liu Z. Gangadharan A. Bieerkehazhi S. Zhao Y.-Z. Alekseev A. (2019). Function of T Follicular Helper Cells in Anti-tumor Immunity. Am. Assoc. Immnol. 202 (1 Supplement), 138.18.

[B28] Ozenne B. Sørensen A. L. Scheike T. Torp-Pedersen C. Gerds T. A. (2017). riskRegression: Predicting the Risk of an Event Using Cox Regression Models. R. J. 9, 440–460. 10.32614/rj-2017-062

[B29] Protti M. P. De Monte L. (2020). Dual Role of Inflammasome Adaptor ASC in Cancer. Front Cel Dev Biol. 8, 40. 10.3389/fcell.2020.00040 PMC7010858 32117971

[B30] Rébé C. Ghiringhelli F. (2020). Interleukin-1β and Cancer. Cancers (Basel) 12, 1791. 10.3390/cancers12071791 PMC7408158 32635472

[B31] Ritchie M. E. Phipson B. Wu D. Hu Y. Law C. W. Shi W. (2015). Limma powers Differential Expression Analyses for RNA-Sequencing and Microarray Studies. Nucleic Acids Res. 43, e47. 10.1093/nar/gkv007 25605792 PMC4402510

[B32] Saeki N. Usui T. Aoyagi K. Kim D. H. Sato M. Mabuchi T. (2009). Distinctive Expression and Function of Four GSDM Family Genes (GSDMA-D) in normal and Malignant Upper Gastrointestinal Epithelium. Genes Chromosomes Cancer 48, 261–271. 10.1002/gcc.20636 19051310

[B33] Shi J. Gao W. Shao F. (2017). Pyroptosis: Gasdermin-Mediated Programmed Necrotic Cell Death. Trends Biochem. Sci. 42, 245–254. 10.1016/j.tibs.2016.10.004 27932073

[B34] Shi J. Zhao Y. Wang Y. Gao W. Ding J. Li P. (2014). Inflammatory Caspases Are Innate Immune Receptors for Intracellular LPS. Nature 514, 187–192. 10.1038/nature13683 25119034

[B35] Sung H. Ferlay J. Siegel R. L. Laversanne M. Soerjomataram I. Jemal A. (2021). Global Cancer Statistics 2020: GLOBOCAN Estimates of Incidence and Mortality Worldwide for 36 Cancers in 185 Countries. CA Cancer J. Clin. 71, 209–249. 10.3322/caac.21660 33538338

[B36] Szklarczyk D. Gable A. L. Lyon D. Junge A. Wyder S. Huerta-Cepas J. (2019). STRING V11: Protein-Protein Association Networks with Increased Coverage, Supporting Functional Discovery in Genome-wide Experimental Datasets. Nucleic Acids Res. 47, D607–d613. 10.1093/nar/gky1131 30476243 PMC6323986

[B37] Team R. C. Bivand R. Carey V. J. Debroy S. Eglen S. Guha R. (2020). Package ‘foreign’. Available at: https://cran.r-project.org/web/packages/foreign.

[B38] Therneau T. M. Lumley T. (2015). Package ‘survival. R. Top. Doc. 128, 28–33.

[B39] Tsuchiya K. (2020). Inflammasome-associated Cell Death: Pyroptosis, Apoptosis, and Physiological Implications. Microbiol. Immunol. 64, 252–269. 10.1111/1348-0421.12771 31912554

[B40] Van Opdenbosch N. Lamkanfi M. (2019). Caspases in Cell Death, Inflammation, and Disease. Immunity 50, 1352–1364. 10.1016/j.immuni.2019.05.020 31216460 PMC6611727

[B41] Vande Walle L. Lamkanfi M. (2016). Pyroptosis. Curr. Biol. 26, R568–r572. 10.1016/j.cub.2016.02.019 27404251

[B42] Walter W. Sánchez-Cabo F. Ricote M. (2015). GOplot: an R Package for Visually Combining Expression Data with Functional Analysis. Bioinformatics 31, 2912–2914. 10.1093/bioinformatics/btv300 25964631

[B43] Wang B. Yin Q. (2017). AIM2 Inflammasome Activation and Regulation: A Structural Perspective. J. Struct. Biol. 200, 279–282. 10.1016/j.jsb.2017.08.001 28813641 PMC5733693

[B44] Wang L. Li X. Zhao L. Jiang L. Song X. Qi A. (2021). Identification of DNA-Repair-Related Five-Gene Signature to Predict Prognosis in Patients with Esophageal Cancer. Pathol. Oncol. Res. 27, 25. 10.3389/pore.2021.596899 PMC8262199 34257547

[B45] Wang S. S. Liu W. Ly D. Xu H. Qu L. Zhang L. (2019). Tumor-infiltrating B Cells: Their Role and Application in Anti-tumor Immunity in Lung Cancer. Cel Mol Immunol. 16, 6–18. 10.1038/s41423-018-0027-x PMC6318290 29628498

[B46] Xia X. Wang X. Cheng Z. Qin W. Lei L. Jiang J. (2019). The Role of Pyroptosis in Cancer: Pro-cancer or Pro-"host. Cell Death Dis. 10, 650. 10.1038/s41419-019-1883-8 31501419 PMC6733901

[B47] Yu G. Wang L. G. Han Y. He Q. Y. (2012). clusterProfiler: an R Package for Comparing Biological Themes Among Gene Clusters. OMICS 16, 284–287. 10.1089/omi.2011.0118 22455463 PMC3339379

[B48] Yu P. Zhang X. Liu N. Tang L. Peng C. Chen X. (2021). Pyroptosis: Mechanisms and Diseases. Signal. Transduct Target. Ther. 6, 128. 10.1038/s41392-021-00507-5 33776057 PMC8005494

[B49] Zeng Q. Fu J. Korrer M. Gorbounov M. Murray P. J. Pardoll D. (2018). Caspase-1 from Human Myeloid-Derived Suppressor Cells Can Promote T Cell-independent Tumor Proliferation. Cancer Immunol. Res. 6, 566–577. 10.1158/2326-6066.CIR-17-0543 29653983 PMC9336537

[B50] Zhang Z. Chen C. Fang Y. Li S. Wang X. Sun L. (2021). Development of a Prognostic Signature for Esophageal Cancer Based on Nine Immune Related Genes. BMC Cancer 21, 113. 10.1186/s12885-021-07813-9 33541291 PMC7860013

[B51] Zheng M. Kanneganti T. D. (2020). The Regulation of the ZBP1-NLRP3 Inflammasome and its Implications in Pyroptosis, Apoptosis, and Necroptosis (PANoptosis). Immunol. Rev. 297, 26–38. 10.1111/imr.12909 32729116 PMC7811275

[B52] Zhou Z. He H. Wang K. Shi X. Wang Y. Su Y. (2020). Granzyme A from Cytotoxic Lymphocytes Cleaves GSDMB to Trigger Pyroptosis in Target Cells. Science 368. 10.1126/science.aaz7548 32299851

[B53] Zhu L. Yang F. Wang L. Dong L. Huang Z. Wang G. (2021). Identification the Ferroptosis-Related Gene Signature in Patients with Esophageal Adenocarcinoma. Cancer Cel Int 21, 124. 10.1186/s12935-021-01821-2 PMC7891153 33602233

