# Supplementary material for: Predicting the Prognosis of Esophageal Adenocarcinoma by a Pyroptosis-Related Gene Signature
Source: Front Pharmacol. 2021 Nov 18;12:767187. doi: 10.3389/fphar.2021.767187 (PMC8637127; doi:10.3389/fphar.2021.767187)
Supplement: Supplementary file 2 [file Table1.DOCX]

| **Gene ID** | **Full-name** |
| --- | --- |
| AIM2 | absent in melanoma 2 |
| APIP | APAF1 Interacting Protein |
| CASP1 | cysteine-aspartic acid protease-1 |
| CASP3 | cysteine-aspartic acid protease-3 |
| CASP4 | cysteine-aspartic acid protease-4 |
| CASP5 | cysteine-aspartic acid protease-5 |
| CASP6 | cysteine-aspartic acid protease-6 |
| CASP8 | cysteine-aspartic acid protease-8 |
| CASP9 | cysteine-aspartic acid protease-9 |
| DHX9 | DExH-Box Helicase 9 |
| ELANE | elastase, neutrophil expressed |
| GPX4 | glutathione peroxidase 4 |
| GSDMA | gasdermin A |
| GSDMA2 | gasdermin A2 |
| GSDMA3 | gasdermin A3 |
| GSDMB | gasdermin B |
| GSDMC | gasdermin C |
| GSDMC2 | gasdermin C2 |
| GSDMC3 | gasdermin C3 |
| GSDMC4 | gasdermin C4 |
| GSDMD | gasdermin D |
| GSDME | gasdermin E |
| GZMA | granzyme A |
| GZMB | granzyme B |
| GZMC | granzyme C |
| GZMD | granzyme D |
| GZME | granzyme E |
| GZMF | granzyme F |
| GZMG | granzyme G |
| GZMN | granzyme N |
| IL18 | interleukin 18 |
| IL1B | interleukin 1 beta |
| IL6 | interleukin 6 |
| NAIP1 | NLR family apoptosis inhibitory protein 1 |
| NAIP2 | NLR family apoptosis inhibitory protein 2 |
| NAIP5 | NLR family apoptosis inhibitory protein 5 |
| NAIP6 | NLR family apoptosis inhibitory protein 6 |
| NAIP7 | NLR family apoptosis inhibitory protein 7 |
| NLRC4 | NLR family CARD domain containing 4 |
| NLRP1 | NLR family pyrin domain containing 1 |
| NLRP1A | NLR family pyrin domain containing 1A |
| NLRP1B | NLR family pyrin domain containing 1B |
| NLRP2 | NLR family pyrin domain containing 2 |
| NLRP3 | NLR family pyrin domain containing 3 |
| NLRP6 | NLR family pyrin domain containing 6 |
| NLRP7 | NLR family pyrin domain containing 7 |
| NLRP9B | NLR family pyrin domain containing 9B |
| NOD1 | nucleotide binding oligomerization domain containing 1 |
| NOD2 | nucleotide binding oligomerization domain containing 2 |
| PJVK | pejvakin/deafness, autosomal recessive 59 |
| PLCG1 | phospholipase C gamma 1 |
| PRKACA | protein kinase cAMP-activated catalytic subunit alpha |
| PYCARD | PYD and CARD domain containing |
| SCAF11 | SR-related CTD associated factor 11 |
| TIRAP | TIR domain containing adaptor protein |
| TNF | tumor necrosis factor |
| TREM2 | triggering receptor expressed on myeloid cells 2 ... |
| ZBP1 | Z-DNA Binding Protein 1 |
