# Supplementary figures and images for: Predicting the Prognosis of Esophageal Adenocarcinoma by a Pyroptosis-Related Gene Signature
Source: Front Pharmacol. 2021 Nov 18;12:767187. doi: 10.3389/fphar.2021.767187 (PMC8637127; doi:10.3389/fphar.2021.767187)

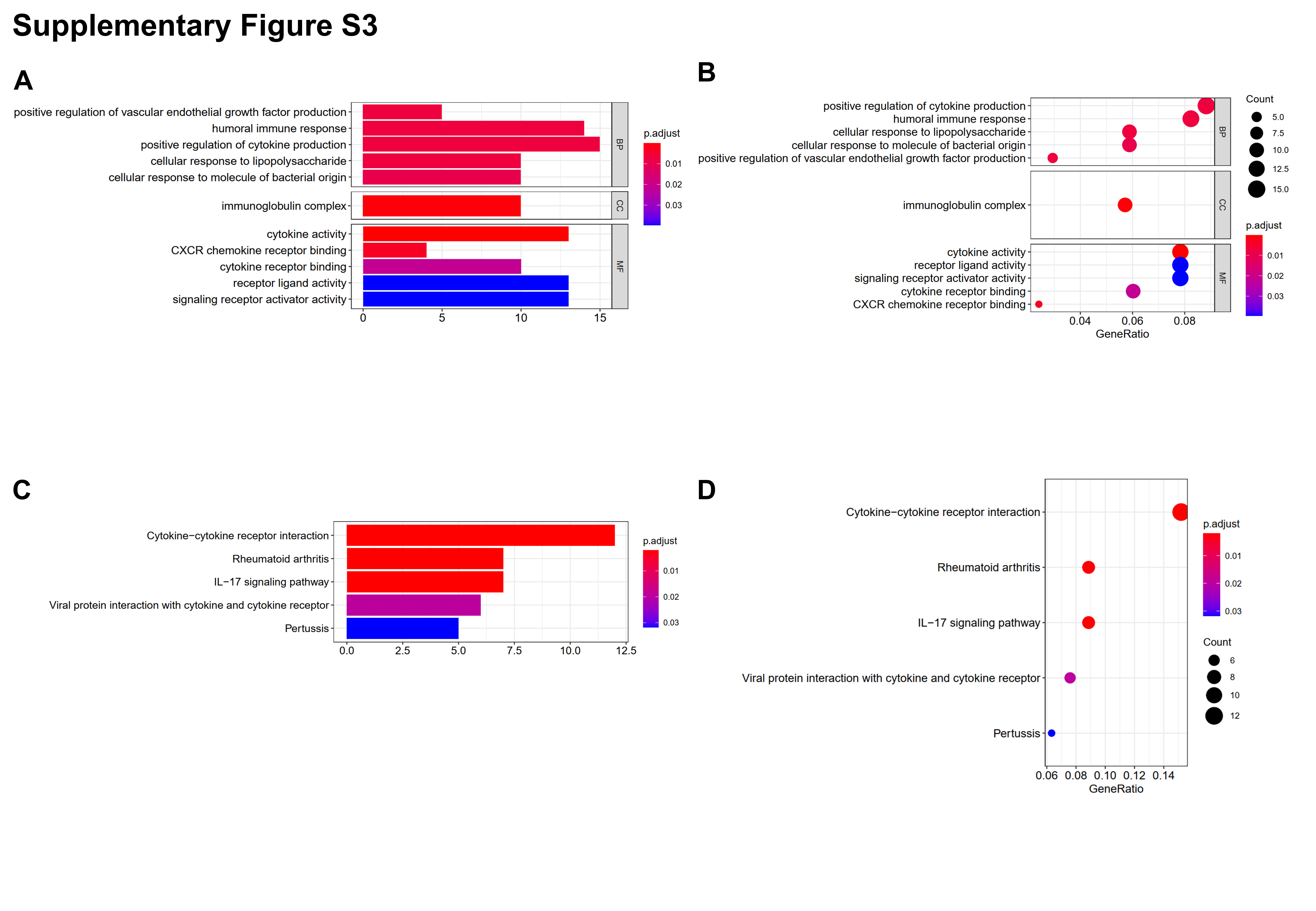

Supplement: Supplementary file 3 [file Image3.TIF]

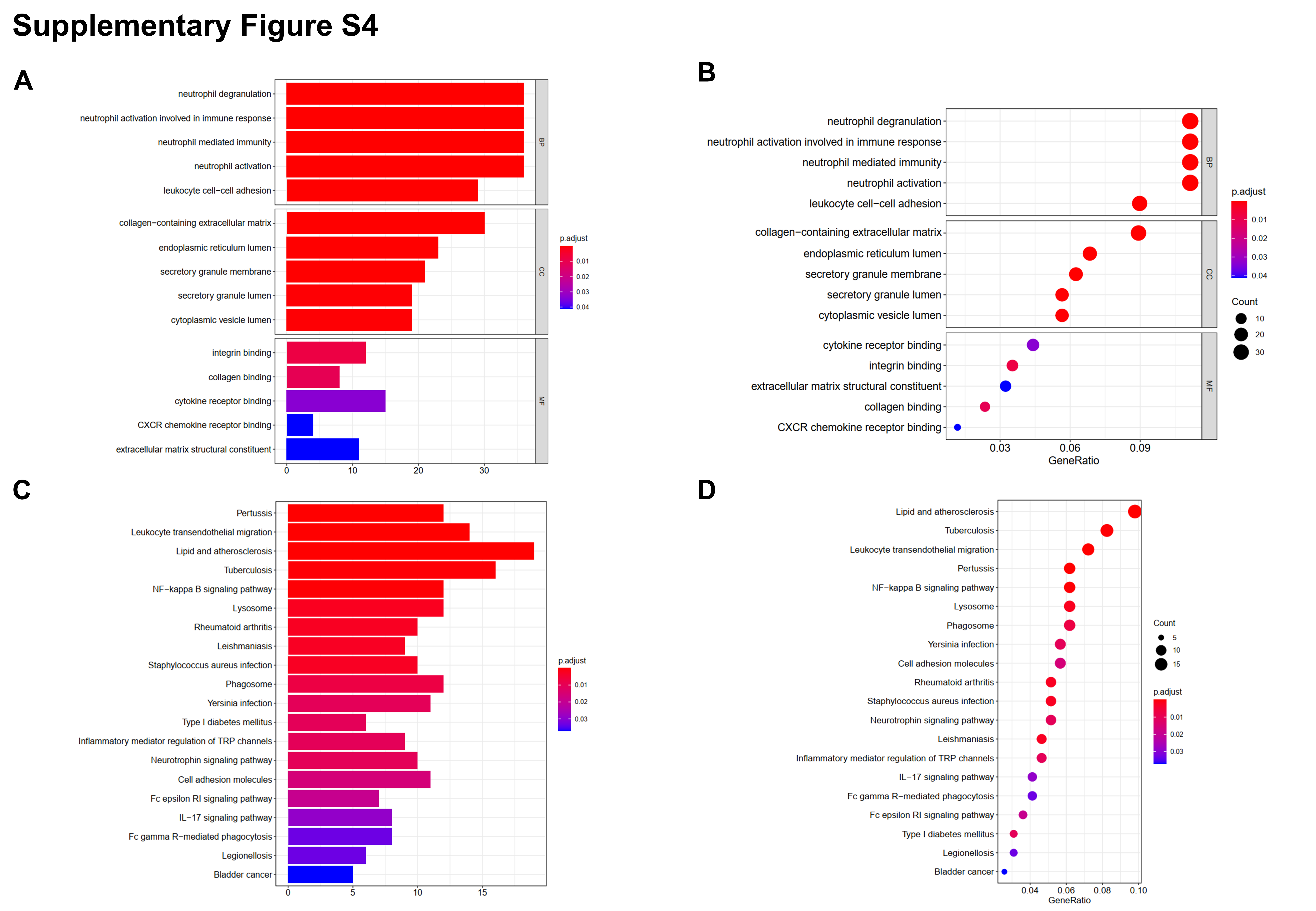

Supplement: Supplementary file 4 [file Image4.TIF]

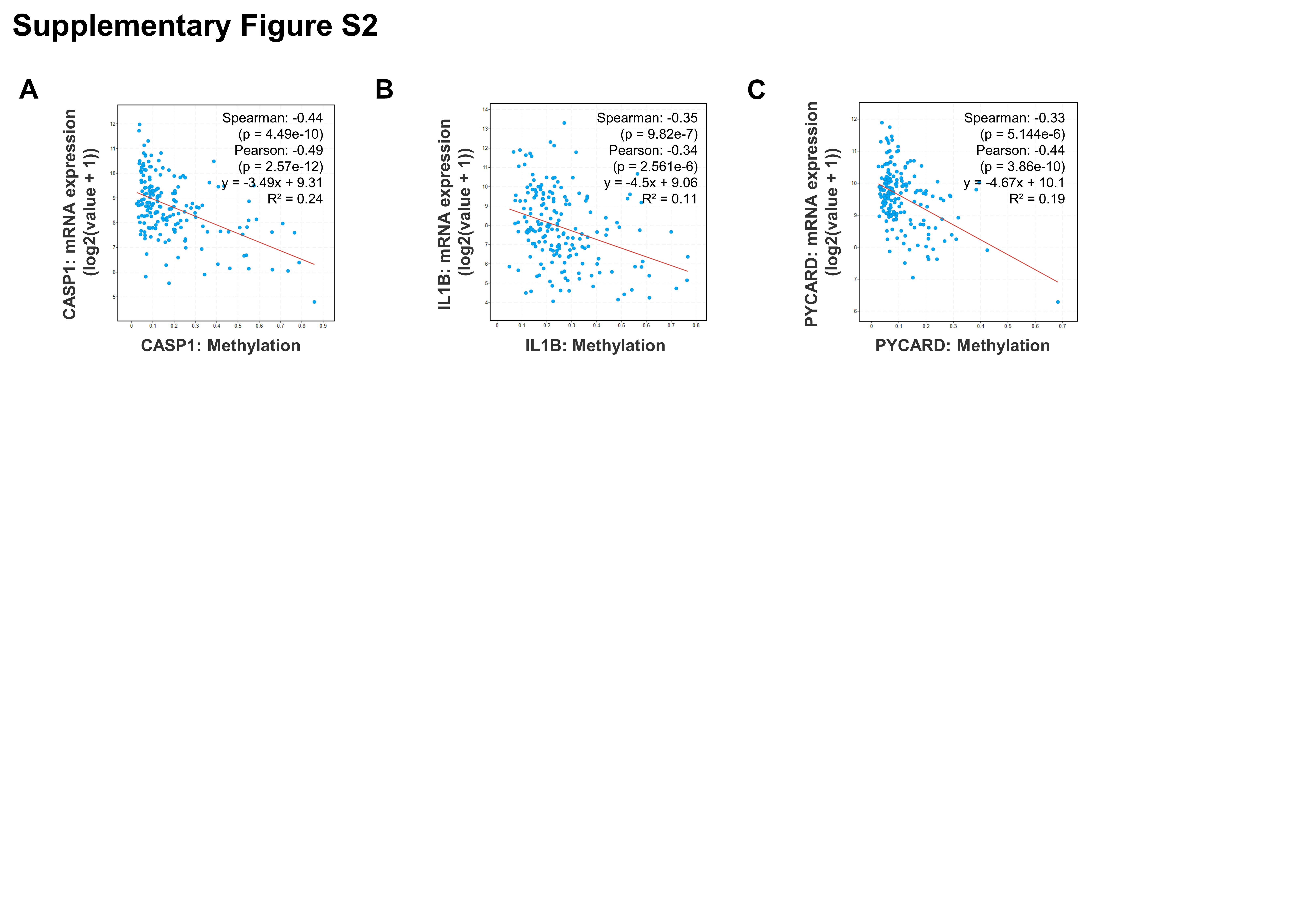

Supplement: Supplementary file 5 [file Image2.TIF]

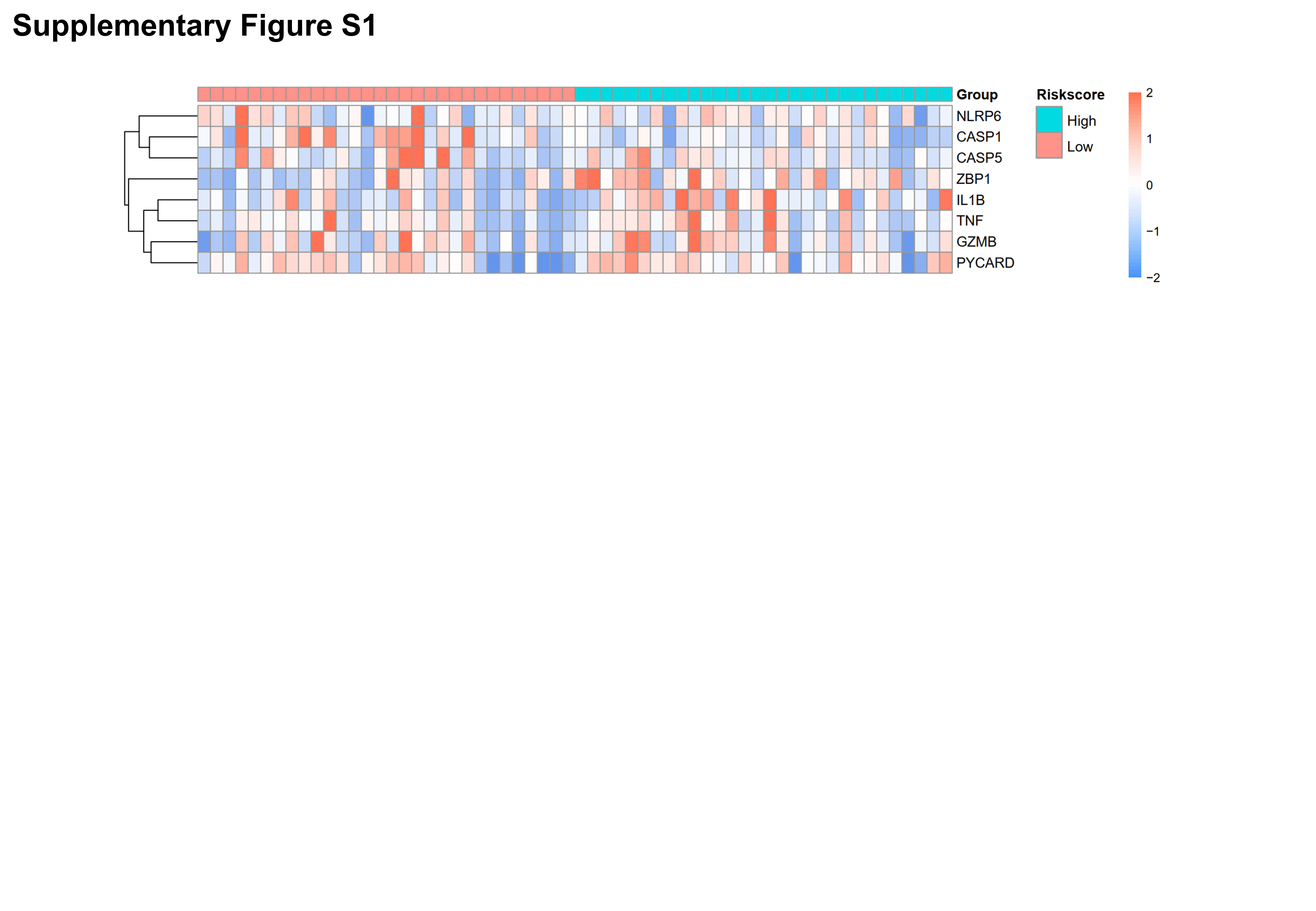

Supplement: Supplementary file 6 [file Image1.TIF]

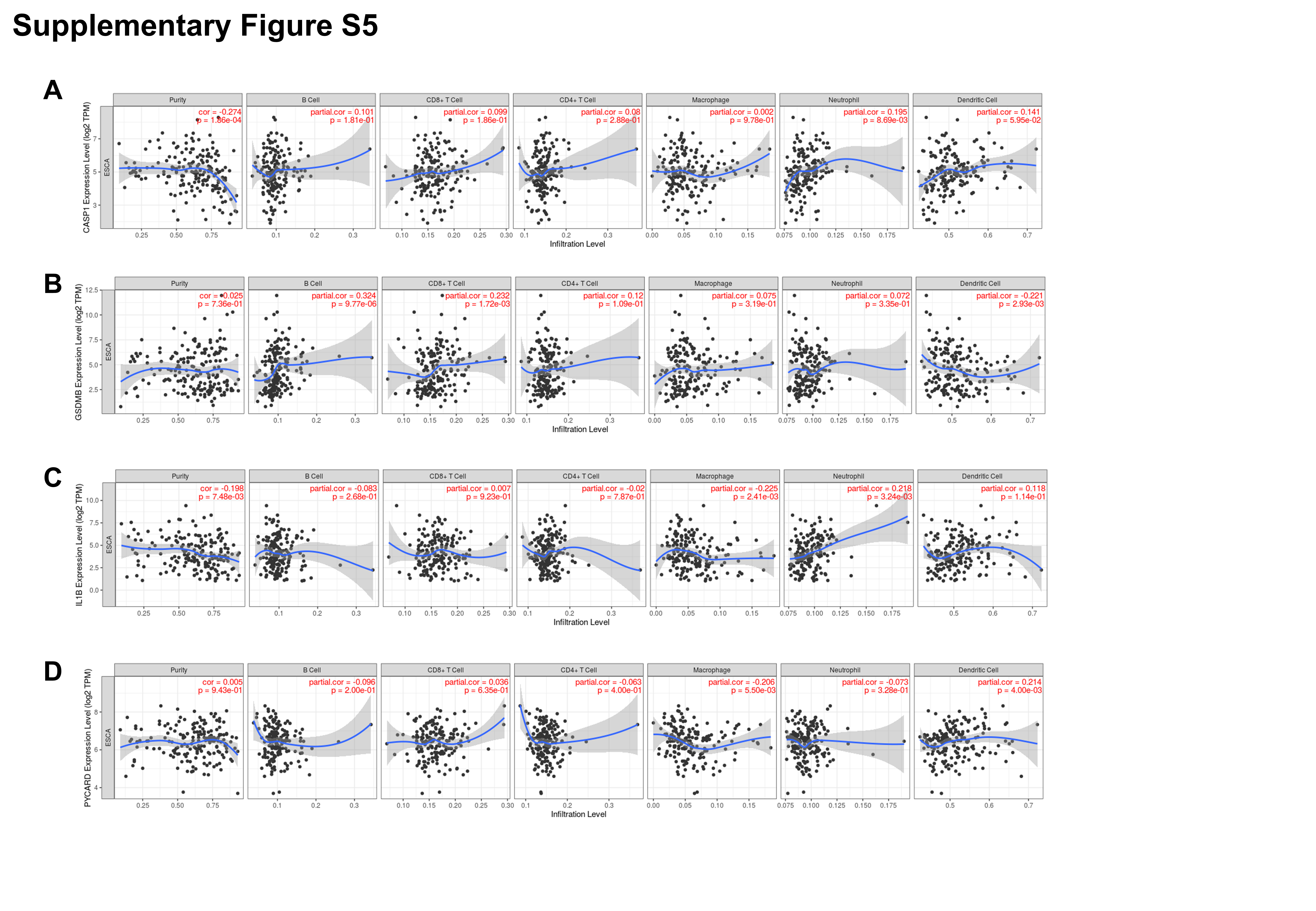

Supplement: Supplementary file 8 [file Image5.TIF]
