# Supplementary material for: Predicting the Prognosis of Esophageal Adenocarcinoma by a Pyroptosis-Related Gene Signature
Source: Front Pharmacol. 2021 Nov 18;12:767187. doi: 10.3389/fphar.2021.767187 (PMC8637127; doi:10.3389/fphar.2021.767187)
Supplement: Supplementary file 7 [file Table2.DOCX]

**Supplementary Table S2.** Clinicopathologic characteristics of esophageal adenocarcinoma (EAC) patients with complete data for further analysis from TCGA and GSE13898 cohorts.

| **Variables** | **TCGA-EAC (n=67)** | | **GSE13898 (n=46)** | |
| --- | --- | --- | --- | --- |
|  | **N** | **%** | **N** | **%** |
| **Age (years)** |  |  |  |  |
| ≤60 | 21 | 31.3 | 27 | 58.7 |
| >60 | 46 | 68.7 | 19 | 41.3 |
| **Gender** |  |  |  |  |
| Female | 10 | 14.9 | 4 | 8.7 |
| Male | 57 | 85.1 | 42 | 91.3 |
| **Stage** |  |  |  |  |
| Stage I | 11 | 16.4 | 20 | 43.5 |
| Stage II | 22 | 32.8 | 20 | 43.5 |
| Stage III | 29 | 43.3 | 6 | 13.0 |
| Stage IV | 5 | 7.5 | 0 | 0.0 |
